# Implicit Flow Cytometric Diagnosis of Classic Hodgkin Lymphoma Using CD3^+^CD4^+^CD26^−^ T‐Cells

**DOI:** 10.1002/jcla.25096

**Published:** 2024-09-05

**Authors:** Curtis Gravenmier, Jinming Song, Haipeng Shao

**Affiliations:** ^1^ Department of Pathology H. Lee Moffitt Cancer Center Tampa Florida USA

**Keywords:** CD26, CD4, classic Hodgkin lymphoma, flow cytometry, T‐cells

## Abstract

**Background:**

Flow cytometry is not routinely performed in clinical laboratories for the diagnosis of classic Hodgkin lymphoma (CHL).

**Methods:**

Fourteen cases of CHL and 132 cases of the control group were studied by 10‐color flow cytometry, with markers including CD3, CD4, CD7, CD8, and CD26, as well as calculated parameters such as the CD4:CD8 ratio, percent CD3^+^CD4^+^CD26^−^ T‐cells of CD3^+^CD4^+^ T‐cells, percent CD3^+^CD4^+^CD26^−^ T‐cells of total events, CD7 coefficient of variation among CD3^+^CD4^+^CD26^−^ T‐cells, and CD7 median fluorescence intensity of CD3^+^CD4^+^CD26^−^ T‐cells relative to CD3^+^CD8^+^ T‐cells.

**Results:**

CHL cases showed a median percent CD3^+^CD4^+^CD26^−^ of CD3^+^CD4^+^ T‐cells of 72.3% with range from 41.1% to 94.4%, median percent CD3^+^CD4^+^CD26^−^ T‐cells of total events of 17.4% with range from 4.6% to 52.5%, CD7 coefficient of variation among CD3^+^CD4^+^CD26^−^ T‐cells less than 100%, and CD7 median fluorescence intensity of CD3^+^CD4^+^CD26^−^ T‐cells relative to CD3^+^CD8^+^ T‐cells of 1.7 with range from 0.4 to 3.5. In the control group, every entity showed some degree of overlap with CHL in terms of these parameters. A “Hodgkin score” was thus constructed to enhance separation of CHL from other entities. A threshold Hodgkin score of 15.35 achieved a sensitivity of 78.6% and specificity of 96.2% in the diagnosis of CHL. Incorporating the Hodgkin score into a simple algorithm raises the specificity to 100%.

**Conclusion:**

In this study, we used flow cytometry to demonstrate increased CD3^+^CD4^+^CD26^−^ T‐cells in CHL, and derived a Hodgkin score for the diagnosis of CHL.

## Graphical abstract

We used flow cytometry to demonstrate increased CD3^+^CD4^+^CD26^−^ T‐cells in classic Hodgkin lymphoma (CHL), and designed a “Hodgkin score” to enhance separation of CHL from other reactive entities or T‐cell lymphomas. A threshold Hodgkin score of 15.35 achieved a sensitivity of 78.6% and specificity of 96.2% in the diagnosis of CHL.

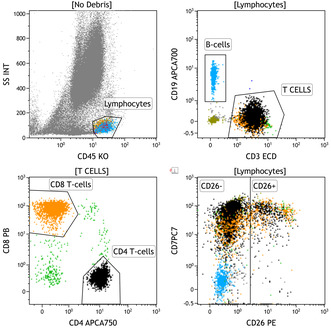

## 1 Introduction

Classic Hodgkin lymphoma (CHL) is characterized histologically by small numbers of Hodgkin and Reed‐Sternberg (HRS) cells dispersed in a rich mixed inflammatory background including small mature lymphocytes, eosinophils, plasma cells, and histiocytes [1]. The HRS cells originate from germinal center B‐cells with functional immunoglobulin gene rearrangement but defective B‐cell gene expression, and show a distinctive immunophenotype with expression of CD30, CD15, weak PAX5, and MUM1, and lack of CD45 and other B‐cell markers. The diagnosis of CHL is established by the identification of HRS cells in an appropriate background and demonstration of the characteristic immunophenotype by immunohistochemical staining. At present, needle core biopsy is frequently used to obtain tissue samples for the diagnosis of lymphoma. This practice presents a challenge in the diagnosis of CHL, as the neoplastic HRS cells are generally outnumbered by non‐neoplastic cells and show a patchy distribution. As a result, HRS cells may not be adequately sampled in needle core biopsies. Excisional biopsy rectifies most non‐diagnostic needle core biopsies but may be difficult or impractical, especially for deep lymph nodes.

Flow cytometry is typically not used for CHL diagnosis in clinical practice, in part due to difficulty obtaining sufficient HRS cells in a sample. Nevertheless, several studies have succeeded in using flow cytometry to phenotype HRS cells with antibody panels including CD30, CD15, CD20, CD40, CD64, and CD95 [2, 3, 4, 5]. HRS cells show high forward and side scatter, characteristic expression of CD30, CD15, CD40 and CD95, and usually no expression of CD20 and CD64. The flow cytometric diagnosis of CHL was shown to have high sensitivity and specificity in small biopsies and FNA material in one study [5]. Another study found frequent expression of CD123 in HRS cells [6]. Unfortunately, most clinical laboratories have not validated the flow cytometry markers used in these studies. Other investigators have attempted to use flow cytometry to aid the diagnosis of CHL by phenotyping T‐cells in the inflammatory background. Findings such as an increased CD4:CD8 ratio, increased CD7 and CD71 expression by CD4^+^ T‐cells, and increased T‐regulatory cells (CD4^+^CD25^+^CD152^+^) have been reported [7, 8, 9, 10, 11, 12].

In this investigation, we used flow cytometry to analyze background CD4^+^ T‐cells from 14 cases of CHL and 132 control cases which included a variety of T‐cell lymphoproliferative disorders and reactive conditions. We demonstrated increased CD3^+^CD4^+^CD26^−^ T‐cells in CHL and increased variability in CD7 expression among T‐cell lymphoproliferative disorders relative to non‐neoplastic T‐cells. A value we call the Hodgkin score was calculated based on the expression of two widely implemented markers (CD7 and CD26) and proved suitable to detect CHL without isolating HRS cells. The Hodgkin score was derived from nondimensional quantities (i.e., unitless values such as percentage) to enhance reproducibility. Lastly, an algorithm incorporating the Hodgkin score was formulated to rule in CHL when specific criteria are met. The algorithm achieved 78.6% sensitivity and 100% specificity for CHL, offering a reliable indicator for most cases in clinical practice.

## 2 Materials and Methods

### 2.1 Patient Selection

Lymph node, bone marrow, and solid organ biopsy reports with a histologic diagnosis of CHL rendered at Moffitt Cancer Center between August 2017 and August 2022 were reviewed to identify cases on which a standardized 10‐color flow cytometry T‐cell panel was performed to evaluate T‐cells in the mixed inflammatory background. CHL diagnosis was based on the 2017 revised WHO Classification of Tumors of Haematopoietic and Lymphoid Tissues [1]. Control flow cytometry cases utilizing the same panel were selected from reports released between January and August 2022. The tissue source, final pathologic diagnosis, and flow cytometry interpretation were independently reviewed by two hematopathologists (HS, CG). This study was approved by the University of South Florida Institutional Review Board.

### 2.2 Flow Cytometry

All samples were analyzed fresh and stained with antibodies within 24 h after collection. Aliquots of 50 μL were incubated in the dark for 15 min at room temperature with combinations of 10 monoclonal antibodies conjugated to Pacific Blue (PB), Krome Orange (KO), fluorescein isothiocyanate (FITC), phycoerythrin (PE), R‐phycoerythin‐Texas Red‐X (ECD), phycoerythrin‐cyanine 5.5 (PC5.5), phycoerythrin‐cyanin 7 (PC7), allophycocyanin (APC), allophycocyanin‐alexa fluor 700 (APC‐A700), and allophycocyanin‐alexa fluor 750 (APC‐A750). Red blood cells were lysed with BD FACS lysing solution (BD Biosciences, San Jose, CA), then nucleated cells were resuspended in phosphate buffered saline containing 2% paraformaldehyde. A standardized 10‐color T‐cell panel (PB, KO, FITC, PE, ECD, PC5.5, PC7, APC, APC‐A700, APC‐A750) was utilized in all specimens, consisting of one stained tube (CD8, CD45, CD2, CD26, CD3, CD5, CD7, CD30, CD19, CD4). All antibodies were obtained from Beckman Coulter (Brea, CA). Up to 100,000 events were acquired until 5000 lymphocytes were collected on a Gallios flow cytometer (Beckman Coulter).

**FIGURE 1:**
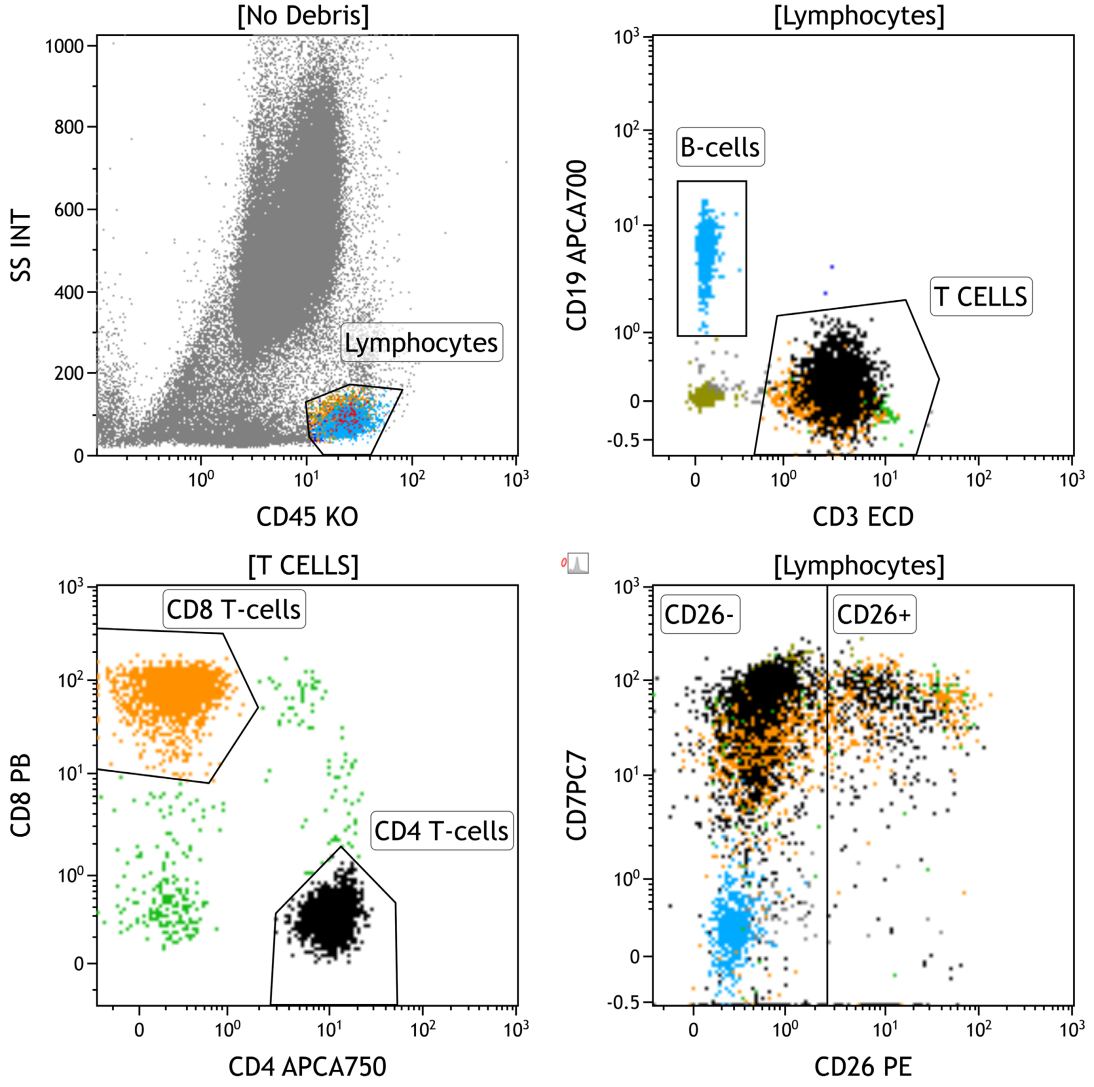
Flow cytometry gating strategy. Four dot‐plots were used to isolate CD4 and CD8 positive T‐cells, then calculate the percent CD3^+^CD4^+^CD26^−^ of CD3^+^CD4^+^ T‐cells, percent CD3^+^CD4^+^CD26^−^ T‐cells of total events, CD7 coefficient of variation among CD3^+^CD4^+^CD26^−^ T‐cells, CD7 median fluorescence intensity of CD3^+^CD4^+^CD26^−^ T‐cells relative to CD3^+^CD8^+^ T‐cells, and Hodgkin score. Depending on the tissue source, either B‐cells or neutrophils were used as an internal reference for CD26 expression. Neutrophils were essentially absent from lymph node specimens involved by CHL, so B‐cells (blue) typically served as the CD26^−^ reference population in lymph nodes. The converse was true in bone marrow specimens, which lack B‐cells but contain plentiful neutrophils. The plots in this figure correspond to CHL involving a lymph node.

Listmode files were analyzed using Kaluza version 2.1 (Beckman Coulter, Brea, CA). First, CD4^+^ and CD8^+^ T‐cells were defined using interdependent polygonal gates drawn on three separate dot‐plots: side scatter versus CD45, CD19 versus CD3, and CD8 versus CD4. Next, CD4^+^ T‐cells were plotted against CD7 and CD26. Then, real‐time color coding was applied to either B‐cells or neutrophils to create an internal reference for CD26 expression. B‐cells were used as an internal reference in specimens without sufficient neutrophils (e.g., lymph node). See Figure 1 for representative dot‐plots which illustrate the gating strategy. At last, the percent CD3^+^CD4^+^CD26^−^ of CD3^+^CD4^+^ T‐cells, percent CD3^+^CD4^+^CD26^−^ T‐cells of total events, CD7 coefficient of variation among CD3^+^CD4^+^CD26^−^ T‐cells, and CD7 median fluorescence intensity of CD3^+^CD4^+^CD26^−^ T‐cells relative to CD3^+^CD8^+^ T‐cells were calculated manually or using the appropriate statistical function in Kaluza (coefficient of variation).

### 2.3 Hodgkin Score

**FIGURE 2:**
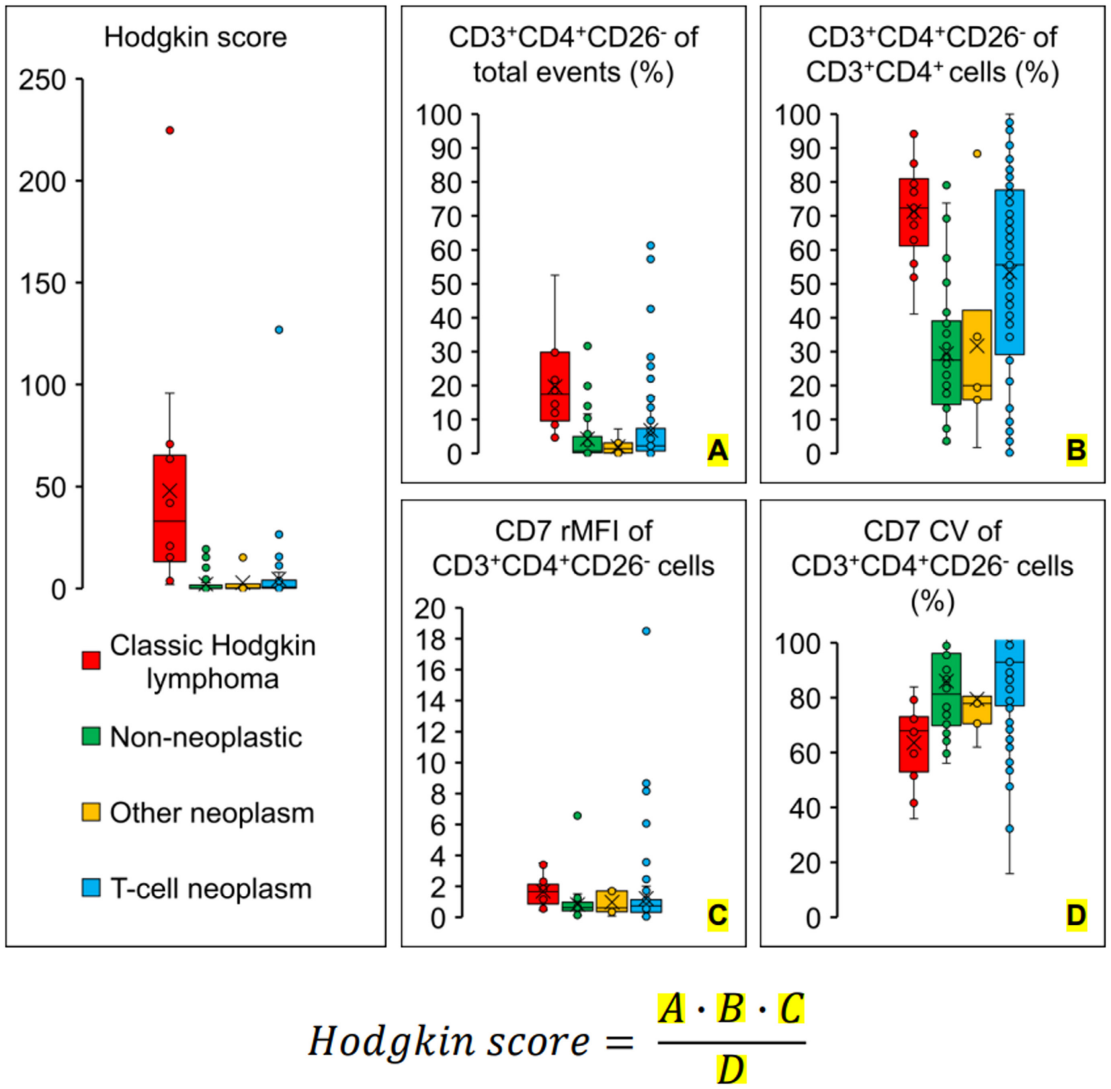
Comparison of flow cytometry parameters between CHL and other entities. CHL cases showed increased CD3^+^CD4^+^CD26^−^ T‐cells, modestly elevated CD7 median fluorescence intensity of CD3^+^CD4^+^CD26^−^ T‐cells relative to CD3^+^CD8^+^ T‐cells, and low CD7 coefficient of variation among CD3^+^CD4^+^CD26‐ T‐cells, resulting in a high Hodgkin score. CV, coefficient of variation; RMFI, relative median fluorescence intensity with respect to CD3^+^CD8^+^ T‐cells.

The Hodgkin score (Figure 2) was constructed from the percent CD3^+^CD4^+^CD26^−^ of CD3^+^CD4^+^ T‐cells (A), percent CD3^+^CD4^+^CD26^−^ T‐cells of total events (B), CD7 median fluorescence intensity of CD3^+^CD4^+^CD26^−^ T‐cells relative to CD3^+^CD8^+^ T‐cells (C), and CD7 coefficient of variation among CD3^+^CD4^+^CD26^−^ T‐cells (D) as follows:

$$\text{Hodgkin score}=\frac{A\times B\times C}{D}$$

**FIGURE 3:**
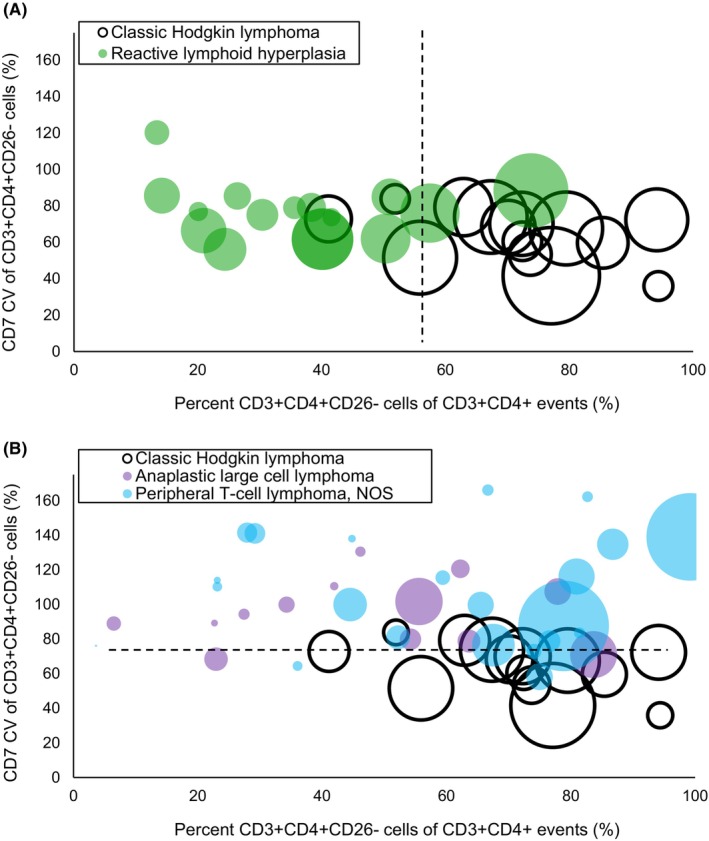
Bubble plots help visualize separation of CHL from non‐CHL cases. Bubble size is proportional to the percent CD3^+^CD4^+^CD26^−^ cells of total events. The prevalence of CD3^+^CD4^+^CD26^−^ cells is the primary basis for distinguishing CHL from reactive lymphadenopathy (A). Most CHL cases have a relatively large CD3^+^CD4^+^CD26^−^ T‐cell population, presumably due to rosetting of neoplastic cells by this T‐cell subset. Meanwhile, the coefficient of variation of CD7 expression by CD3^+^CD4^+^CD26^−^ cells is the primary basis for distinguishing CHL from T‐cell lymphoma (B). Most CHL cases have a relatively low CD7 coefficient of variation. Anaplastic large cell lymphoma and peripheral T‐cell lymphoma were chosen for comparison because, like CHL, these entities may express CD30, involve deep lymph nodes, and be obscured by non‐neoplastic inflammatory cells.

The rationale for this form is that HRS‐bound T‐cells are CD3^+^CD4^+^CD26^−^ and should be enriched in cases of CHL. Although CD4^+^ T‐cell lymphomas also frequently show diminished or absent CD26 expression, we noticed greater variability in CD7 expression among CD4^+^ T‐cell lymphomas than among CD3^+^CD4^+^CD26^−^ T‐cells in CHL (Figure 3). Therefore, the CD7 coefficient of variation was included in the denominator to lower the Hodgkin score for CD4^+^ T‐cell lymphomas, which might otherwise be indistinguishable from CHL background T‐cells.

## 3 Results

Fourteen CHL cases were identified that had been subjected to the required flow cytometry panel, including 8 nodular sclerosis subtype, 2 mixed cellularity subtype, and 4 unspecified subtype due to scant tissue. An enlarged lymph node was the tissue source in 12 of 14 cases and sampling was performed by either fine needle aspiration with needle core biopsy (*n* = 7) or excisional biopsy (*n* = 5). An additional 2 CHL cases were diagnosed through posterior iliac crest bone marrow biopsy. The median CD4:CD8 ratio among CHL was 5.8 and ranged from 1.3 to 60.3, although most cases (12 of 14) showed a CD4:CD8 ratio between 2 and 10. The median percent CD3^+^CD4^+^CD26^−^ of CD3^+^CD4^+^ T‐cells was 72.3% with range from 41.1% to 94.4%, and many cases showed distinct CD26^+^ and CD26^−^ T‐cell subpopulations. The median percent CD3^+^CD4^+^CD26^−^ T‐cells of total events was 17.4% with range from 4.6% to 52.5%. The CD7 coefficient of variation among CD3^+^CD4^+^CD26^−^ T‐cells was invariably less than 100%, and the CD7 median fluorescence intensity of CD3^+^CD4^+^CD26^−^ T‐cells relative to CD3^+^CD8^+^ T‐cells was 1.7 with range from 0.4 to 3.5. The median Hodgkin score among CHL was 33.0 and ranged from 2.0 to 224.6.

The control group comprised 132 additional cases representing a variety of neoplastic and non‐neoplastic diagnoses. There were 78 cases of T‐cell lymphoma including anaplastic large cell lymphoma (*n* = 15), mycosis fungoides/Sezary syndrome (*n* = 25), peripheral T‐cell lymphoma not otherwise specified (*n* = 23), angioimmunoblastic T‐cell lymphoma (*n* = 8), adult T‐cell leukemia/lymphoma (*n* = 2), T‐cell prolymphocytic leukemia (*n* = 3), and T‐cell large granular lymphocytic leukemia (*n* = 2). A small number of B‐cell non‐Hodgkin lymphomas including follicular lymphoma and diffuse large B‐cell lymphoma had been examined using the T‐cell flow cytometry panel and were also included in the data set (*n* = 4). Two cases of myelodysplastic syndrome were similarly included. The remaining cases covered a variety of benign conditions categorized as reactive lymphadenopathy (*n* = 16), benign large granular lymphocyte proliferations (*n* = 6), granulomatous inflammation (*n* = 3), normal or allogeneic transplanted bone marrow (*n* = 18), and other inflammatory diagnoses (*n* = 5). Tissue sources consisted of lymph node (*n* = 67), bone marrow (*n* = 55), skin (*n* = 14), spleen/tonsil (*n* = 3), lung (*n* = 3), mesentery (*n* = 2), duodenum (*n* = 1), and cortical bone (*n* = 1).

Every entity in the control group showed some degree of overlap with CHL in terms of the measured parameters. Mycosis fungoides/Sezary syndrome and reactive lymphadenopathy presented the most significant overlap and, therefore, became the primary obstacles to crafting an effective Hodgkin score. Angioimmunoblastic T‐cell lymphoma and peripheral T‐cell lymphoma not otherwise specified often displayed increased CD4^+^CD26^−^ T‐cells, but in other respects were straightforward to distinguish from CHL. Table 1 provides the median and range for each parameter in all entities. Figure 2 is a graphical representation (box and whisker diagram).

**TABLE 1:** Summary of flow cytometry analysis and key data.

Diagnosis	Hodgkin score	% CD3^+^CD4^+^CD26^−^ of total cells	% CD3^+^CD4^+^CD26^−^ of CD3^+^CD4^+^ T‐cells	CD7 rMFI of CD3^+^CD4^+^CD26^−^ T‐cells	CD7 CV of CD3^+^CD4^+^CD26^−^ T‐cells
	*Median* (*Range*)	*Median* (*Range*)	*Median* (*Range*)	*Median* (*Range*)	*Median* (*Range*)
CHL (*n = 14*)	33.0 (2.0–224.6)	17.4 (4.6–52.5)	72.3 (41.1–94.4)	1.7 (0.4–3.5)	68.0 (36.0–83.9)
ALCL (*n = 15*)	0.3 (0.01–13.2)	2.0 (0.1–16.7)	46.2 (6.5–83.7)	0.7 (0.1–2.0)	99.9 (68.4–235.2)
PTCL, NOS (*n = 23*)	0.7 (0.003–126.9)	3.2 (0.03–61.3)	66.7 (3.6–99.2)	0.5 (0.1–18.5)	110.3 (58.2–201.3)
AILT (*n = 8*)	1.29 (0.02–6.9)	3.2 (0.4–16.2)	28.7 (4.6–53.7)	0.8 (0.4–1.7)	79.4 (63.1–110.1)
MF/SS (*n = 25*)	1.5 (0.01–27.6)	5.2 (0.2–42.6)	70.6 (9.4–97.6)	0.7 (0.1–8.7)	99.1 (47.7–206.8)
ATLL (*n = 2*)	1.9 (1.4–2.3)	2.2 (1.4–3.0)	94.1 (92.1–96.1)	0.9 (0.4–1.3)	82.6 (77.0–88.2)
T‐PLL (*n = 3*)	0.1 (0.01–0.1)	0.02 (0.01–0.1)	70.0 (50.0–100.0)	1.1 (0.8–1.3)	68.6 (15.9–101.2)
T‐LGLL (*n = 2*)	13.2 (0.0–26.4)	2.2 (0.01–4.4)	19.9 (0.2–39.5)	4.4 (0.7–8.2)	42.9 (32.3–53.5)
B‐NHL (*n = 4*)	1.0 (0.0–15.2)	1.6 (0.0–7.2)	31.1 (19.5–88.4)	1.2 (0.1–1.8)	76.0 (62.0–80.6)
RLH (*n = 16*)	2.3 (0.1–19.3)	7.5 (1.9–31.6)	37.0 (13.4–73.8)	0.8 (0.3–1.3)	76.5 (56.1–120.2)
LGL (*n = 6*)	0.1 (0.0–0.9)	0.5 (0.03–1.9)	14.7 (0.3–40.6)	1.0 (0.1–1.5)	89.0 (78.5–201.7)
Granulomatous inflammation (*n = 3*)	0.01 (0.0–0.2)	0.1 (0.04–0.8)	8.3 (5.1–31.7)	0.7 (0.4–1.1)	64.2 (59.7–90.2)
MDS (*n = 2*)	0.01 (0.0–0.01)	0.1 (0.09–0.2)	8.8 (1.8–15.8)	0.5 (0.4–0.6)	91.9 (70.6–113.2)
Normal BM (*n = 13*)	0.02 (0.0–0.5)	0.2 (0.1–1.8)	17.7 (3.1–35.3)	0.5 (0.1–1.1)	78.6 (61.6–129.6)
Allo‐SCT (*n = 5*)	0.1 (0.0–1.1)	0.3 (0.04–1.5)	37.0 (3.7–69.2)	0.5 (0.4–1.5)	95.5 (76.6–112.6)
Other (*n = 5*)	0.05 (0.04–2.3)	0.5 (0.3–3.1)	34.4 (14.6–79.1)	0.5 (0.1–6.6)	95.8 (77.9–174.9)

Abbreviations: AILT, angioimmunoblastic T cell lymphoma; ALCL, anaplastic large cell lymphoma; Allo‐SCT, allogeneic hematopoietic stem cell transplanted bone marrow; ATLL, adult T‐cell leukemia/lymphoma; BM, bone marrow; B‐NHL, B‐cell non‐Hodgkin lymphoma; CHL, classic Hodgkin lymphoma; CV, coefficient of variation; LGL, reactive or clonal large granular lymphocytosis; MDS, myelodysplastic syndrome; MF/SS, mycosis fungoides/Sezary syndrome; MFI, relative mean fluorescence intensity with respect to CD3^+^CD8^+^ T‐cells; PTCL, NOS: peripheral T cell lymphoma NOS; RLH, reactive lymphoid hyperplasia; T‐LGLL, T cell large granular lymphocytic leukemia; T‐PLL, T prolymphocytic leukemia.

**FIGURE 4:**
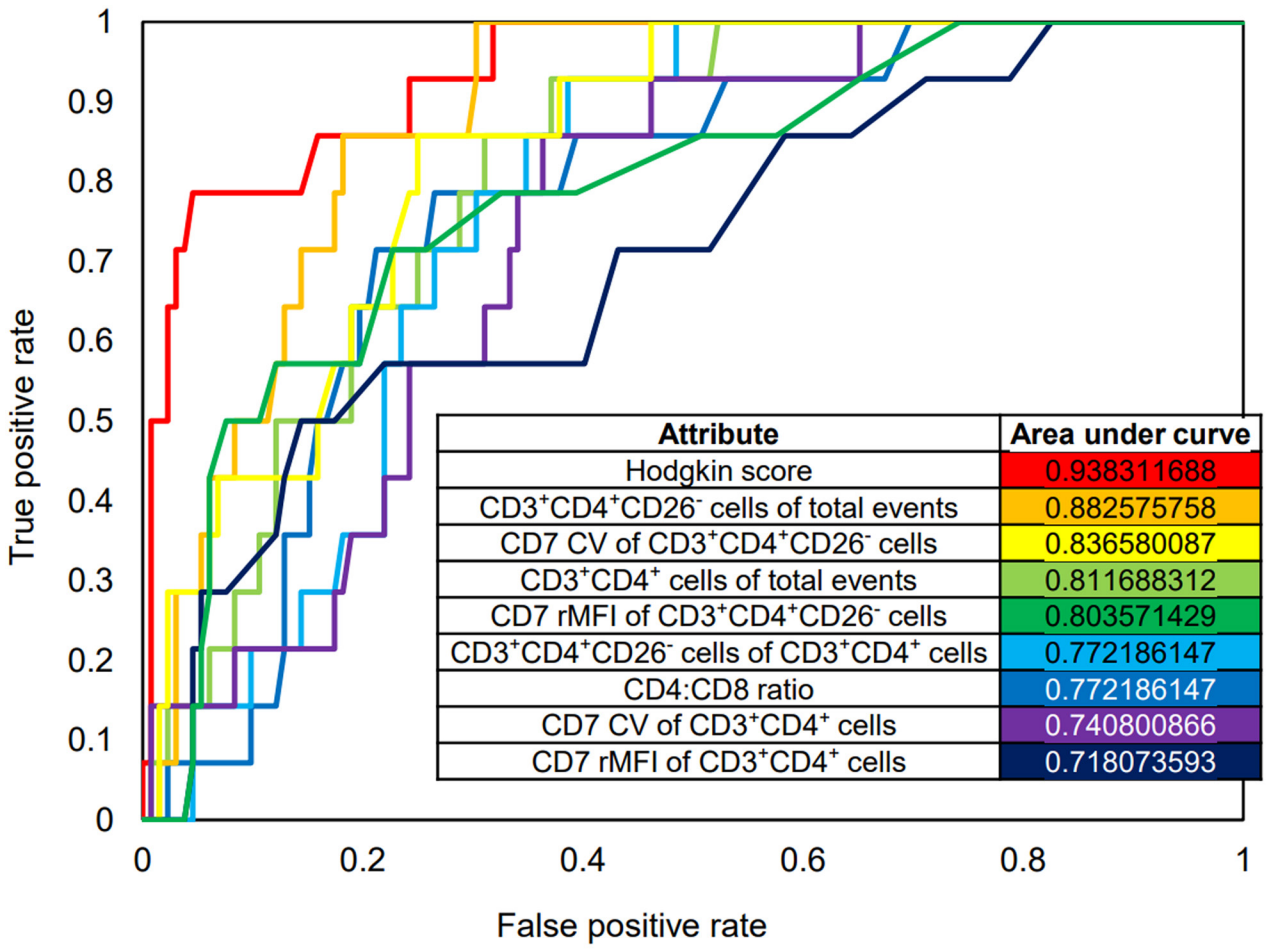
Diagnostic performance of the Hodgkin score. A receiver operating characteristic was made to visualize the diagnostic performance of the Hodgkin score and related flow cytometry parameters as predictors of CHL. The Hodgkin score was superior to other parameters and achieved a sensitivity of 78.6% and specificity of 96.2% if greater than 15.35. CV, coefficient of variation; RMFI, relative median fluorescence intensity with respect to CD3^+^CD8^+^ T‐cells.

**FIGURE 5:**
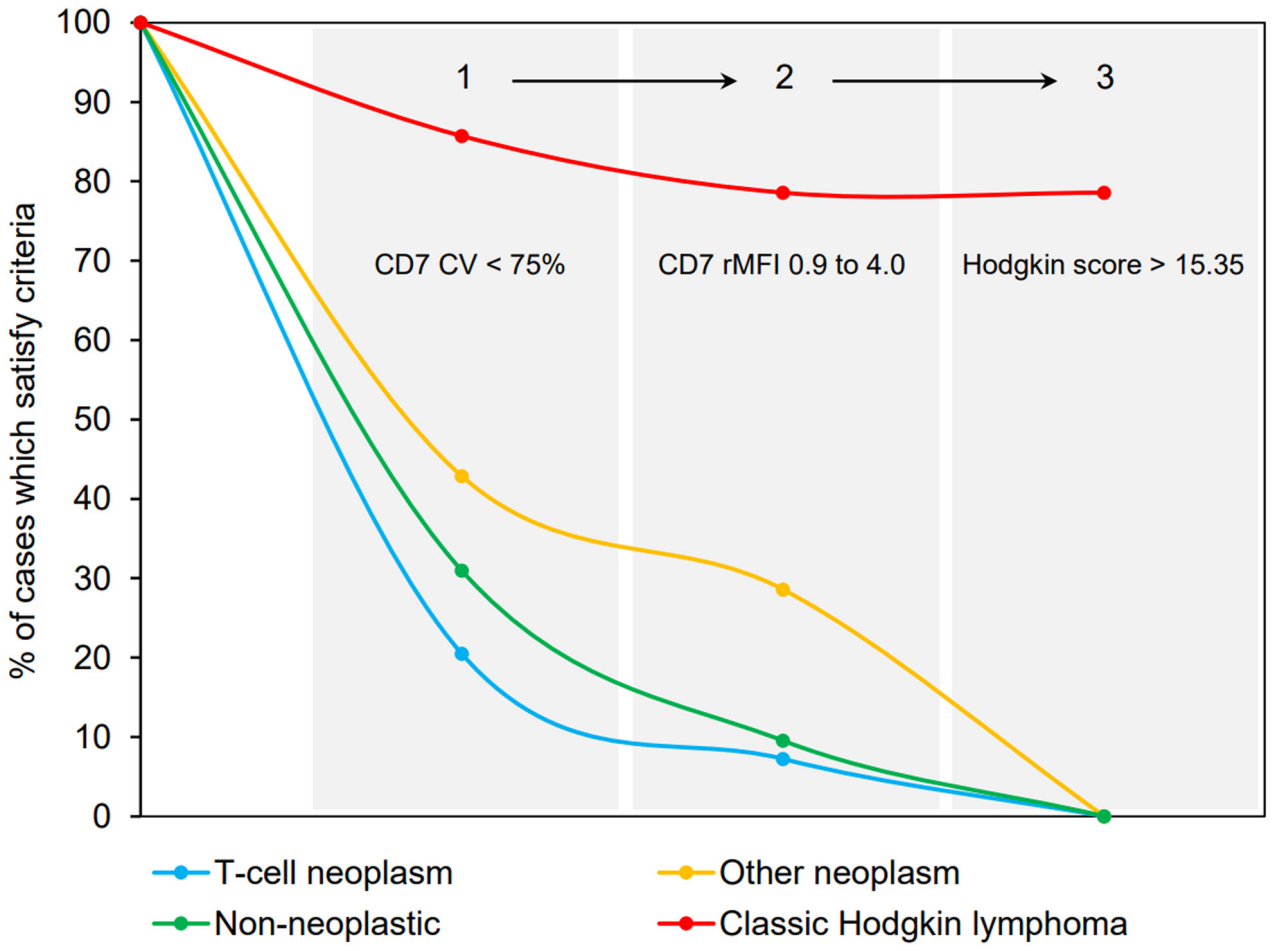
Stepwise application of the Hodgkin score to rule in CHL. A simple algorithm was proposed to incorporate the Hodgkin score into clinical practice. If ambiguous microscopic findings are encountered in a setting suspicious for CHL, the following three flow cytometry criteria were 100% specific for CHL in our data set: (1) Hodgkin score greater than 15.35, (2) CD7 coefficient of variation of CD3^+^CD4^+^CD26^−^ T‐cells <75%, and (3) CD7 relative median fluorescence intensity of CD3^+^CD4^+^CD26^−^ T‐cells relative to CD3^+^CD8^+^ T‐cells between 0.9 and 4.0. These criteria preserve a 78.6% sensitivity. CV, coefficient of variation among CD3^+^CD4^+^CD26^−^ T‐cells; RMFI, median fluorescence intensity of CD3^+^CD4^+^CD26^−^ T‐cells relative to CD3^+^CD8^+^ T‐cells.

The diagnostic performance of each flow cytometry parameter and the Hodgkin score was assessed using a receiver operating characteristic (Figure 4). The area under the curve (AUC) was 0.938 for the Hodgkin score, 0.772 for percent CD3^+^CD4^+^CD26^−^ of CD3^+^CD4^+^ T‐cells, 0.883 for percent CD3^+^CD4^+^CD26^−^ T‐cells of total events, 0.804 for CD7 median fluorescence intensity of CD3^+^CD4^+^CD26^−^ T‐cells relative to CD3^+^CD8^+^ T‐cells, and 0.837 for the CD7 coefficient of variation among CD3^+^CD4^+^CD26^−^ T‐cells. A few related parameters including the CD4:CD8 ratio were also examined. A threshold Hodgkin score of 15.35 achieved a sensitivity of 78.6% and specificity of 96.2% in our data set. Incorporating the Hodgkin score into a 3‐step algorithm raised the specificity to 100% (Figure 5).

## 4 Discussion

CHL is difficult to diagnose in small specimens with relatively few neoplastic HRS cells. In the CHL inflammatory background, CD4^+^ T‐cells form rosettes around HRS cells, guided by interactions between CD2/LFA‐1 on T‐cells and CD54/CD58 on HRS cells [13]. These T‐cells co‐express bright CD7, CD45RO, and lymphocyte activation markers including CD38 and HLA‐DR. CD26 is not expressed by the T‐cells, although it is also a marker of lymphocyte activation. In CHL, mRNA profiling of the CD4^+^CD26^−^ T‐cell subset reveals limited cytokine production despite phorbol 12‐myristate 13‐acetate and ionomycin stimulation, and the T‐cells are positive for exhaustion‐associated transcription factors TOX and TOX2 by immunohistochemistry [14, 15]. Together, these features indicate that HRS‐bound T‐cells are antigen experienced lymphocytes which have lost their effector function; likely a manifestation of immune evasion by HRS cells.

CD4^+^CD26^−^ T‐cells are of course not unique to CHL. A similar T‐cell subset forms rosettes around neoplastic B‐cells in nodular lymphocyte predominant Hodgkin lymphoma, and increased CD4^+^CD26^−^ T‐cells can also be observed in lymph nodes with reactive changes. T‐cell lymphomas, especially mycosis fungoides/Sezary syndrome (MF/SS) and adult T‐cell leukemia/lymphoma, sometimes display a CD4^+^CD26^−^ immunophenotype. This feature is widely employed to stage MF/SS per the EORTC cutaneous lymphoma taskforce recommended protocol, which quantitates CD4^+^CD7^−^ and CD4^+^CD26^−^ T‐cells in peripheral blood [16]. For these reasons, CD26 is a validated marker in many clinical flow cytometry laboratories.

Though flow cytometry is capable of directly detecting HRS cells, most clinical laboratories have not implemented the required markers. Attempts to diagnose CHL using flow cytometry characteristics of the inflammatory background have been moderately successful but face limited adoption by pathologists. We used flow cytometry of CD3^+^CD4^+^CD26^−^ T‐cells to derive a value called the Hodgkin score that achieved a sensitivity of 78.6%, specificity of 96.2%, and area under the curve of 0.938 in our data set. The Hodgkin score is equal to the product of three parameters: (1) percent CD3^+^CD4^+^CD26^−^ of CD3^+^CD4^+^ T‐cells, (2) percent CD3^+^CD4^+^CD26^−^ T‐cells of total events, and (3) CD7 median fluorescence intensity of CD3^+^CD4^+^CD26^−^ T‐cells relative to CD3^+^CD8^+^ T‐cells, divided by a fourth parameter: (4) CD7 coefficient of variation among CD3^+^CD4^+^CD26^−^ T‐cells (Figure 2). In isolation, the percent CD3^+^CD4^+^CD26^−^ T‐cells of total events was a strong indicator of CHL, as expected because HRS cells are rosetted by CD3^+^CD4^+^CD26^−^ T‐cells. However, this parameter failed to exclude some cases of anaplastic large cell lymphoma, angioimmunoblastic T‐cell lymphoma, peripheral T‐cell lymphoma not otherwise specified, mycosis fungoides/Sezary syndrome, and reactive lymphadenopathy. In keeping with prior reports, we observed that the CD7 median fluorescence intensity of CD3^+^CD4^+^CD26^−^ T‐cells in CHL is increased relative to T‐cells in these overlapping cases, and therefore included this parameter in the numerator of the Hodgkin score. We further observed that CD7 expression shows greater variability in T‐cell lymphoma than in CD3^+^CD4^+^CD26^−^ T‐cells of CHL and improved the Hodgkin score by including the CD7 coefficient of variation among CD3^+^CD4^+^CD26^−^ T‐cells in its denominator.

Maximal specificity would be required if the Hodgkin score were used to diagnose CHL without direct detection of HRS cells. To this end, we designed a 3‐step algorithm that reaches 100% specificity and maintains 78.6% sensitivity for CHL (Figure 5). The following two criteria exclude 92.8% of T‐cell lymphomas, 90.5% of non‐neoplastic entities, and 71.4% of non‐T‐cell neoplasms in the control group: (1) the CD7 coefficient of variation for CD3^+^CD4^+^CD26^−^ T‐cells <75%, and (2) CD7 median fluorescence intensity of CD3^+^CD4^+^CD26^−^ T‐cells relative to CD3^+^CD8^+^ T‐cells between 0.9 and 4.0. The remaining non‐CHL cases where excluded when the Hodgkin score exceeded 15.35. In our data set, an accurate diagnosis of CHL could be rendered if all three criteria were met.

The main strengths of our approach include the use of widely implemented flow cytometry markers and accurate exclusion of T‐cell lymphomas, which could present with large CD30 positive cells in deep lymph nodes, especially anaplastic large cell lymphoma, angioimmunoblastic T‐cell lymphoma, and peripheral T‐cell lymphoma not otherwise specified. A major weakness of the study, however, is the absence of nodular lymphocyte predominant Hodgkin lymphoma (NLPHL) and T‐cell/histiocyte‐rich large B‐cell lymphoma (THRLBL) from the data set. Unfortunately, flow cytometry panels at our institution are designed with specific entities in mind rather than broad applicability because patients are often referred to us with an established diagnosis. We were unable to identify cases of NLPHL or THRLBL that had been analyzed using the required flow cytometry T‐cell panel. It was difficult to accrue 14 CHL cases for this same reason. It would not be surprising for NLPHL to have extensive overlap with CHL in terms of the parameters used in our study, since the neoplastic B‐cells of both entities are rosetted by CD3^+^CD4^+^CD26^−^ T‐cells. Still, it may be possible to separate NLPHL from CHL by assessing the percent CD4^+^CD8^+^ dual positive T‐cells of total events, which is known to be elevated in the NLPHL inflammatory background.

## Conflicts of Interest

The authors declare no conflicts of interest.

## Data Availability

The data that support the findings of this study are available from the corresponding author upon reasonable request.
